# Quality of Life amongst Older Brazilians: A Cross-Cultural Validation of the CASP-19 into Brazilian-Portuguese

**DOI:** 10.1371/journal.pone.0094289

**Published:** 2014-04-16

**Authors:** Fábia M. Lima, Martin Hyde, Holendro Singh Chungkham, Clarice Correia, Alexsandra Siqueira Campos, Marília Campos, Moacir Novaes, Jerson Laks, Kátia Petribu

**Affiliations:** 1 Universidade de Pernambuco, Recife, Pernambuco, Brazil; 2 Stress Research Institute, Stockholm University, Stockholm, Sweden; 3 Researcher 2 Conselho Nacional de Pesquisa (CNPq), Institute of Psychiatry, Universidade Federal do Rio de Janeiro, Rio de Janeiro, Brasil; Johns Hopkins Bloomberg School of Public Health, United States of America

## Abstract

**Introduction:**

As population ageing becomes a global phenomenon the need to understand the quality of life of older people around the world has become increasingly salient. The CASP-19 is a well established measure of quality of later life. The scale is composed of 19 items which map onto the four domains of control (C), Autonomy (A), Self-Realisation (S) and Pleasure (P). It has already been translated to 12 languages and has been used in a number of national and international studies. However use of the scale outside of Europe has been very limited. The objective of this study was to translate and evaluate the use of the CASP-19 amongst older Brazilians.

**Methods:**

The CASP-19 was translated from English to Portuguese, back-translated and submitted to an analysis of equivalence by a committee of judges. The scale was then administered to a sample of community dwelling older people in Recife, Brazil (n = 87), and tested for psychometric properties. The Control and Pleasure domains exhibited good internal consistency. By removing one item from each of the Autonomy and Self Realisation domains their internal consistency was improved.

**Results:**

The mean age of the sample was 75.6±0.7 years, subjects were mainly female (52.9%), white (52.9%), who lived without a partner (54%), and had a monthly income varying from USD 340.00 to USD 850.00. Translation and cross-cultural adaptation permitted good understanding and applicability of final version. Psychometric analyses revealed that the removal of two items improved the internal consistency of the Autonomy and Pleasure domains. Confirmatory factor analyses suggest that a 16 item, four factor, model best fits the data.

**Conclusion:**

In this small exploratory study the CASP-19 Brazil demonstrated good psychometric properties. It was easy to use for both participants and researchers. Hopefully future studies in Brazil will employ the scale so that more direct cross national comparisons can be made with older people in Europe and the US.

## Introduction

Like many middle-income countries the Brazilian population is ageing rapidly. This makes the need to develop suitable measures of quality of life (QoL) in later life in Brazil a pressing matter. Total fertility in Brazil in the early 1950s was 6.15 infants per 1,000 women. By 2005-2010 this had dropped to 1.9 infants. At the same time life expectancy at birth has risen from 50.9 years to 70.9 years. Consequently the proportion of the Brazilian population that is aged 60 years and over has risen from 4.9 per cent in 1950 to 10.3 per cent today and is expected to rise to nearly one-third by 2050 [1], [2] Thus, today there are nearly 21 million Brazilians aged 60 years and over. Over half of those in this age range are women, 55 per cent are White and almost two-thirds are the main income earner in the household. The majority, 66 per cent, are already retired, 12 per cent still receive very low wages, and about one third have low educational status. Nearly half have a chronic illness and 54 per cent of the elderly with more than 75 years of age suffer from more than one chronic disorder [3]. Yet this is still a relatively ‘young-old’ population with those aged 80 years and over only making up 1.5 per cent of the present population [4]. This is similar to other countries in the region [5]–[8]. Nonetheless the pace and extent of population ageing in Brazil raises a number of potential issues about the present and future QoL for older people in the country. In order to ensure that policy makers, health practitioners, charities and individuals themselves can ‘add life to years’ and not just ‘years to life’ it is crucial to develop reliable measures of QoL for use in this age group.

### Quality of life in later life

Quality of life (QoL) in later life has become a major global policy and research issue. In the Madrid Plan of Action the UN clearly identifies the promotion of well-being as a key goal for national and international bodies [9]. This requires a common understanding and comparative measures of quality of life in order to ensure that differences in QoL are not a product of measurement (or more general methodological) differences. There is also a need to assess QoL in different cultures and countries, not only for local purposes but also to assess cross-cultural issues which might be of use in understanding certain different characteristics across various cultures and settings. Although talking more broadly about (health related) QoL, rather than amongst the older population, Skevington [10] identifies three reasons why cross national measures of QoL are needed; i) for designing cross-cultural measures is to serve evidence based medicine in the systematic monitoring of outcomes from multinational clinical trials, ii) to enable comparisons to be made about QoL in different cultural or social groups and iii) to provide theoretical insights into whether QoL is a universal or relativist concept. To this one can add that it is important to understand whether the factors that affect QoL in later life are the same in different countries. All of which has prompted an increasing interest in developing means to evaluate QoL that are specific to this age range [10]–[13]. There has also been move to develop broader approaches measuring QoL that take into consideration more recent approaches of active and positive aging instead of the former approach which focused mainly on physical aging and functional decline [14]–[16].

### CASP-19 in cross-cultural research

The scale that was chosen for the present study was the CASP-19 [17], [18]. It was felt that it met all the desired criteria. As with other scales it covers the positive and beneficial aspects of aging rather than just on the medical and social care that have been seen to typify the aging process [18]–[20]. However, it also has a clear theoretical foundation and the assessment is independent from health conditions as well as from some other factors that may influence QoL. It is a 19-item self-rated questionnaire which covers four inseparable and non-hierarchical life domains; Control, Autonomy, Self-Realization and Pleasure [17], [18], [21]. It has proved to be a quick, objective, and multidimensional instrument with good psychometric properties. The scale has been used in a number of national and cross-national studies in over 20 countries. It has been included in the English Longitudinal Study of Ageing (ELSA) [22], the Survey of Health, Aging and Retirement in Europe (SHARE) [23], the Health and Retirement Survey (HRS) [24], the Health, Alcohol and Psychosocial factors in Eastern Europe (HAPIEE) [25], the British Household Panel Survey [21], the GAZEL study [26], [27] and the CONSTANCE study [28]. These qualities have made the CASP-19 one of the key instruments to evaluate QoL amongst older people. It has shown to be associated with socio-economic position [29]–[32], financial difficulties [33], cognitive function [34], [35], physiological status [20], [36] and engagement in socially productive activities [27], [37]–[40].

Yet there has been only limited use of the scale amongst non-European populations. Amit and Litwin [41] used data from SHARE-Israel to study differences in QoL amongst immigrants in Israel. For this the scale was translated into Arabic and Hebrew. The findings suggest that CASP works well with non-European populations. However a large majority of the respondents had migrated from Europe, notably the Former Soviet Union countries. Also, although Israel is geographically in Asia it is often considered to share a number of cultural similarities with Europe. The scale has also been used in one study in Africa. The results from study of QoL amongst retired Nigerian academics show that the scale performed relatively well. The scale as a whole displayed reasonable internal consistency and good convergent validity with life satisfaction scales [42]. Conversely the scale was dropped from first wave of the Japanese Study of Ageing and Retirement (J-STAR) because it was felt that the some of the domains of control and autonomy were too Western and not applicable to Japanese notions of QoL [43]. However the scale is being validated in a pilot study in Japan and is it may be included in future waves of J-STAR.

However despite the widespread use of the CASP there have been relatively few studies that have tested the psychometric properties of the scale. In the first published study to do this Wiggins and colleagues [21] conducted a mix of exploratory and confirmatory factor analyses on the scale in one English and two British studies. Their results revealed that a 3-factor 12-item model provided the best fit for the data. In this model the Control and Autonomy domains did not appear as distinct from one another. Instead the remaining items from the original domains of loaded on to a new domain that was labeled ‘Control and Autonomy.’ In a smaller study of residents of a British retirement community Sim and colleagues [44] employed a similar approach. They also found little evidence to support the original 4-factor model. However their analyses did support the modified 12-item model proposed by Wiggins et al [21]. Two more recent studies from outside of the UK have, however, shown rather different results. Analyses of the CASP 19 in a representative sample of the Irish population aged 50 years and over failed to support either the original 4-factor, 19-item, model or a 3-factor, 12-item, model. Through the application of a series of modification indices the authors arrive at a 9-item, 2-factor model. In a similar manner to the 3-factor model the Control and Autonomy domains are merged. However, unlike the previous models, the remaining items in the Pleasure and Self-Realisation domains are also merged [45]. Finally a validation study of the Mandarin Chinese version of the CASP scale amongst participants in a health examination in Taiwan revealed a 5-factor model [46]. Although the study confirmed the original 4 factors, albeit with reduced items in the control and autonomy domains, they identified an additional factor that they labeled ‘Participation’. This factor comprised a mix of the control and autonomy items but seemed to form a distinct domain separate from the original domains.

These results suggest that more cross-national validation of the scale is required in general and in particular for use with non-European population. The aim of this study is to provide a cross-cultural adaptation and to measure the psychometric properties of CASP-19 in Brazilian Portuguese.

## Methods

The validation study was carried out in two steps. First, the CASP-19 was translated and culturally adapted into Portuguese. This process was performed according to the four stages described by Guillemin (1995) [54]. Then, the psychometric properties of the instrument were evaluated by application to community dwelling older people in Recife, Pernambuco, Brazil. The total sample included in this study comprised 87 older people with a mean age of 75.6±0.7 years. The elderly were identified and invited to participate in the survey by convenience sampling by a trained census or a community health agent working in the area, who scheduled an interview with the researcher. The sociodemographic characteristics of variables were expressed as absolute or relative frequency distributions (table 1). The participants were mostly Caucasian (52.9%), women (52.9%) and living alone (54%). The socio-demographic data are presented in table 1. Subjects were interviewed from September 2008 to February 2009.

**Table 1:** Socio-demographic characteristics of the sample.

Characteristic socio-demographic	n	%
**Age range (years)**		
65–69	16	18.4
70–79	47	54.0
80–97	24	27.6
**Gender**		
Male	41	47.1
Female	46	52.9
**Ethnicity (self –rated)**		
White	46	52.9
Afro Descendant	5	5.7
Brown/Mullato	36	41.4
**Marital Status**		
Married	40	46.0
Unmarried	47	54.0
**Living arrangements**		
Alone	13	14.9
With partner	40	46.0
Persons living with friends or relatives	34	39.1
**Education**		
None	16	18.4
1–4 years	32	36.8
5–8 years	25	28.7
>9 years	14	16,1
**Family income (minimum wage)**		
≤1	19	21.8
2 to 5	50	57.5
5 to 10	13	14.9
>10	5	5.7
**Participation in social activities**		
**Leisure**		
Yes	38	43.7
No	49	56.3
**Religious**		
Yes	72	82.8
No	15	17.2
**Social Groups**		
Yes	22	25.3
No	65	74.7
**Physical Activities**		
Yes	25	28.7
No	62	71.3

(n = 87).

### Translation and cross-cultural adaptation

Before going into the field the items in the scale were subject to translation and back translation. Two Brazilian bilingual translators (T1, T2) who had no previous contact with the English original CASP-19 independently translated the instrument into Portuguese. The two versions were assessed by a committee constituted of T1, T2, a bilingual observer, a health professional, and the first author of this study. This committee conducted the equivalence analysis, looking at the semantic, idiomatic, cultural and conceptual comparability of the original and the translated items. Following this the items were synthesised into a single instrument. Next, two other translators (T3, T4) who are native English speakers and had no access to any previous version of the CASP-19, independently carried out the back-translation of the Portuguese version into English. Finally, a committee of six judges who were fluent in English (two MDs, one Portuguese teacher, T3, T4 and the first author of this study) assessed the CASP-19 Brazil comparing it to the original English version and to the two back-translated versions. This process resulted in the final version of the CASP-19 Brazil used in the validation process.

### Results of the translation and cross-cultural adaptation

There were three queries raised by the judges on some cultural differences regarding the translations and back-translations. These were on item 4 from the Control domain, item 9 from the Autonomy domain and item 10 from the Pleasure domain. For item 4, the committee preferred the word “tudo”, meaning ‘wholly’, ‘entirely’ or ‘completely’, instead of “coisas”, meaning ‘things’ or ‘articles’, to preserve the meaning from the English original. Indeed, the word “coisas” would render a concept of objects whereas “tudo” would refer more to the idea of life as a whole. Likewise, “shortage of money” was semantically adapted as “falta de dinheiro”, since in Brazil this expression means not only lack of money but also economic difficulties. As for item 10, the committee expressed some doubts on how to define the expression “these days”. The final decision was to change the word “recentemente” (recently), for “hoje em dia” (nowadays, presently), which give the idea of “in the present moment”. The final version is presented in Table 2.

**Table 2:** Item wording for CASP-16 Brazilian Portuguese version.

No.	Original CASP-19 items	Translated CASP-16 Brazil
C1	My age prevents me from doing the things I would like to	Minha idade me impede de fazer as coisas que eu gostaria de fazer
C2	I feel that what happens to me is out of my control	Eu sinto que o que acontece comigo, está fora do meu controle.
C3	I feel free to plan for the future	Eu me sinto livre para planejar o futuro.
C4	I feel left out of things	Eu me sinto excluído de tudo.
A1	I can do the things that I want to do	Eu posso fazer as coisas que eu quero.
A2	Family responsibilities prevent me from doing what I want to do	As responsabilidades familiares me impedem de fazer o que eu quero.
A3	I feel that I can please myself what I can do	Eu me sinto livre para fazer as coisas.
A4	My health stops me from doing the things I want to do	Minha saúde me impede de fazer as coisas que eu quero.
A5	Shortage of money stops me from doing the things I want to do	A falta de dinheiro me impede de fazer as coisas que eu quero.
P1	I look forward to each day	Eu fico animado a cada dia.
P2	I feel that my life has meaning	Eu sinto que minha vida tem sentido.
P3	I enjoy the things that I do	Eu gosto das coisas que faço.
P4	I enjoy being in the company of others	The item has been removed
P5	On balance, I look back on my life with a sense of happiness	The item has been removed
SR1	I feel full of energy these days	Eu me sinto cheio de energia hoje em dia.
SR2	I choose to do things I have never done before	Eu escolho fazer coisas que nunca fiz antes.
SR3	I feel satisfied with the way my life has turned out	The item has been removed
SR4	I feel that life is full of opportunities	Eu sinto que a vida está cheia de oportunidades.
SR5	I feel that the future looks good for me	Eu sinto que o meu futuro parece bom.

### Validation of the Brazilian Portuguese version of CASP-19

Data collection for this validation was done in Recife, the state capital of Pernambuco. This is the second largest city in Northeast Brazil. It has 1,549,980 inhabitants divided into six Political-Administrative Regions. There are a total of 92,824 people aged 65 years and older in Recife. The research was carried out in 28 quarters of the city which, in total, comprise 18,904 people aged 65 years and over from a range of socio-economic groups [1], [2]. The sample size (n = 87) was calculated based on a population of 18,904 older people, an estimated 35 per cent of whom had poor QoL, a 10% precision, design effect = 1 and a significance level = 0.05.The data were collected from October 2008 to January 2009. The data were collected using an interview-administered questionnaire. This contained questions about the respondents' socio-demographic characteristics as well as the CASP-19. The study was approved by the Ethics Committee of the University Hospital Oswaldo Cruz, number 099/08. All the participants signed a consent form before taking part in the study.

## Statistical Analysis

The survey uses the 19-item of the CASP scale. Following Hyde et al [17] each item is scored on a Likert-scale ranging from 0 (often) to 3 (never/almost never). In the initial assessment of the items the skewness, kurtosis and the Cronbach's alpha if item was removed were calculated (table 3) to inform which items would be included in the confirmatory factor analysis (CFA). A number of items, C4, A1, P1-P4 and SR3, displayed excessive skewness (-/+1). This is in line with results found elsewhere [45]. None of the items displayed excessive kurtosis. CFA using structural equation models was carried for the whole sample to test the 4 factor structure proposed in the original study.

**Table 3:** Descriptive statistics for the CASP-19 items.

Items	Mean	Std. Deviation	Skewness	Kurtosis	Item-Total Correlation	Cronbach's Alpha if Item Deleted
C1	1.77	1.14	−0.16	−1.49	0.43	0.63
C2	1.92	1.11	−0.46	−1.24	0.54	0.55
C3	1.98	1.09	−0.67	−0.88	0.47	0.59
C4	2.41	1.01	−1.48	0.72	0.38	0.65
A1	2.28	0.99	−1.18	0.24	0.53	0.57
A2	1.89	1.17	−0.36	−1.47	0.23	0.70
A3	2.34	0.95	−1.25	0.37	0.52	0.58
A4	1.41	1.20	0.19	−1.50	0.48	0.59
A5	1.18	1.10	0.55	−0.99	0.40	0.63
P1	2.32	0.88	−1.21	0.65	0.55	0.50
P2	2.44	0.85	−1.45	1.33	0.51	0.52
P3	2.59	0.80	−1.89	2.60	0.36	0.60
P4	2.70	0.55	−1.71	2.04	0.26	0.64
P5	2.33	1.03	−1.30	0.28	0.32	0.64
SR1	2.26	0.90	−0.95	−0.11	0.56	0.67
SR2	1.25	1.08	0.16	−1.33	0.20	0.80
SR3	2.22	1.04	−1.03	−0.30	0.52	0.68
SR4	2.08	1.07	−0.81	−0.69	0.66	0.62
SR5	2.17	1.06	−0.96	−0.45	0.60	0.65

The goodness of fit for the competing models was evaluated through fit indices [55]. Root mean square error of approximation (RMSEA) incorporates a penalty function for poor model parsimony, expressed by model degrees of freedom. Values under 0.06 are recommended, whereas values above 0.10 indicate poor fit and that the model should be rejected [56]. The comparative fit index (CFI) represents an incremental fit index [55] comparing the hypothesized model to a more restricted nested baseline model; values above 0.95 indicate good fit and values > = 0.90 shows acceptable fit [55]. In the initial factor structure examination, modification indices (MI) were also explored in order to identify parameter misfit. This index reflects how much the overall model chi-square would decrease if a constrained parameter was freely estimated. Possible correlations between indicator measurement errors not previously specified in the model under inspection involving values of a modification index equal or more than 10 would further examined, as well as the magnitude of the corresponding expected parameter changes (EPC) for freely estimated parameters [55].

The overall internal consistency of items in the factor structure was tested by calculating composite reliability (CR) with the relaxation of the assumptions of equal common factor loadings and uncorrelated measurement errors posed in Cronbach's alpha. It has been observed that Cronbach's alpha is a lower bound to reliability and tends to give a grossly underestimated value of reliability in most cases [57]. For a particular factor it is calculated as 
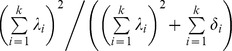
, where *λ_i_* is the standardized loading for the *i^th^* item, and *δ_i_*, the corresponding measurement error from the fitted model. Its value lies between 0 and 1 with value ≥0.70 indicate acceptable internal consistency [58]. The 95% confidence intervals for CR were estimated by bootstrapping with 10000 replications [59].

All statistical tests were carried out in *lavaan* package [60] for R [61] with the use of full information maximum likelihood (FIML) estimation with robust standard errors (MLR) to account for excess skewness and kurtosis using EM algorithm for missing data. Under this robust estimation the difference in χ^2^ does not follow a chi-square distribution, so we use the scaled χ^2^ [62].

## Results

Descriptive statistics, including means, standard deviations, skewness and kurtosis for all the items included in the model are presented in table 3. Many of the items showed low to moderate non-normality. This excess skewness and kurtosis has been taken into account at the analysis stage by adopting robust maximum likelihood estimation (MLR). Before starting the confirmatory factor analysis to evaluate the factor structure of the CASP-19, we checked for the internal consistencies of all the items included in each of the factors to identify the items which do not fit well with the rest of the items in the particular factor. The internal consistency co-efficient in the form of Cronbach's alpha for all the factors are given in Table 4. In this internal consistency exploration, we found that the item-total correlation of the *family responsibilities* item (A2) in the Autonomy sub-domain was only 0.23 which is quite low. The same is also true for the item *never done before* (SR2) of the Self-Realization factor, with an item-total correlation of only 0.20. This shows that these two items do not fit well with the rest of the items in their respective factors. Therefore, we removed these two items from the further modeling of the confirmatory factor structure of the CASP-19. From this point onwards, we have only 17 items available for the CFA modeling.

**Table 4:** Standardized loadings (*λ_i_*), measurement errors (*δ_i_*), factor correlations (*φ_ij_*), composite reliability (*ρ_cr_*) and fit indices from competing CFA models of the CASP-questionnaire.

Items of constructs	Model-I		Model-II		Model-III	
	*λ_i_*	*δ_i_*	*λ_i_*	*δ_i_*	*λ_i_*	*δ_i_*
**Control (CTL)**						
a. *my age prevents*†	0.69*	0.81	0.69*	0.81	0.68*	0.82
b. *out of my control*†	0.75*	0.67	0.75*	0.67	0.75*	0.66
c. *plan for future*	0.63*	0.77	0.63*	0.77	0.64*	0.77
d. *left out of things*†	0.47*	0.78	0.47*	0.78	0.47*	0.78
Composite reliability for **CTL**	0.67 (0.66, 0.70)		0.68 (0.66, 0.70)		0.69 (0.65, 0.72)	
**Autonomy (AUT)**						
f. *I can do things*	0.70*	0.46	0.70*	0.46	0.70*	0.47
g. *I can please myself*	0.64*	0.48	0.64*	0.48	0.64*	0.48
h. *my health stops me*†	0.74*	0.87	0.74*	0.87	0.74*	0.86
i. *shortage of money*†	0.49*	0.95	0.49*	0.95	0.49*	0.95
Composite reliability for **AUT**	0.70 (0.68, 0.73)		0.71 (0.68, 0.73)		0.71 (0.68, 0.73)	
**Self-realization (SER)**						
j. *full of energy*	0.63*	0.40	0.63*	0.40	0.63*	0.41
k. *satisfied with my life*	0.68*	0.61	0.68*	0.60	0.67*	0.62
l. *life is full of opportunities*	0.78*	0.52	0.78*	0.52	0.79*	0.50
m. *future looks good*	0.76*	0.53	0.76*	0.53	0.77*	0.51
Composite reliability for **SER**	0.79 (0.77, 0.81)		0.79 (0.77, 0.81)		0.78 (0.75, 0.81)	
**Pleasure (PLS)**						
n. *forward to each day*	0.69*	0.30	0.67*	0.32	0.67*	0.32
o. *life has meaning*	0.43*	0.52	0.41*	0.54	0.41*	0.53
p. *enjoy the things*	0.40*	0.47	0.41*	0.47	0.42*	0.46
q. *enjoy being in company*	0.08	0.30	-	-	-	-
r. *sense of happiness*	0.51	0.78	0.52*	0.79	0.50*	0.81
Composite reliability for **PLS**	0.64 (0.62, 0.67)		0.65 (0.62, 0.67)		0.65 (0.62, 0.67)	
Item error correlation (item-k 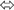 item-r)	-		-		0.26 (0.03, 0.47)	
**Factor correlations** (*φ_ij_*)						
CTL, AUT	0.93 (0.68, 0.95)		0.93 (0.68, 0.94)		0.93 (0.68, 0.94)	
CTL, SER	0.66 (0.49, 0.84)		0.67 (0.50, 0.84)		0.67 (0.49, 0.84)	
CTL, PLS	0.79 (0.60, 0.99)		0.83 (0.59, 0.98)		0.83 (0.58, 0.98)	
AUT, SER	0.71 (0.49, 0.93)		0.71 (0.48, 0.91)		0.70 (0.46, 0.90)	
AUT, PLS	0.73 (0.54, 0.92)		0.77 (0.48, 0.87)		0.77 (0.48, 0.87)	
SER, PLS	0.85 (0.66, 0.98)		0.87 (0.67, 0.98)		0.84 (0.65, 0.96)	
**Goodness of fit indices^#^**						
CFI	0.832		0.891		0.910	
TLI	0.798		0.866		0.899	
RMSEA	0.079		0.066		0.060	
SRMR	0.085		0.075		0.073	
?^2^(*df*)	175.11(113)		135.03(98)		127.56(97)	

*: *p*<0.05; #: scaled for non-normality; figures in parentheses are 95% confidence intervals for estimates; †: reverse coded items.

### Cross-sectional confirmatory factor analysis models

Various alternatives of CFA models were estimated using the data to examine the suitability of the CASP-17 for the data at hand. This stage of analysis is based on a sample of 87 individual included in the survey. As all respondents gave full information on the CASP items there was no item non-response. Therefore all respondents were able to be included in the analyses. The results are shown in Table 4. It is evident that the 17-item four-factor solution (Model-I) showed significant loadings for all the factors included in the model, except for the item *enjoy being in company* of the pleasure factor. The loading of this particular item is extremely low (0.08), which is not significant (*p* = 0.395). This model resulted in a very poor fit index with CFI = 0.832; RMSEA = 0.079; SRMR = 0.085. Therefore, this model is not acceptable [55].

Since Model-I is not acceptable, we fitted an alternative model, Model-II without the item *enjoy being in company* of the pleasure factor (low non-significant loading in Model-I). The resulted loadings are all significant with sizable effect with slight improvement over the previous model Model-I in terms of fit indices (CFI = 0.891; RMSEA = 0.066; SRMR = 0.075). However, Model-II is still below the acceptable level suggested by poor fit indices [55].

At this stage we started looking at the modification indices (MI) suggested from Model-II to see lack of model adequacy. The MI showed that there is an error measurement correlation between *satisfied with my life* (pleasure) and *sense of happiness* (self-realization) would decrease the model's chi-square by 8.28 with an EPC of 0.24. Therefore, with these suggestions an alternative 16-item four-factor model (Model-II) was tested with the error measurement correlation between *satisfied with my life* (pleasure) and *sense of happiness* (self-realization) was confirmed (0.26, significant). The model was adequate with improvement over Model-II (CFI = 0.910, RMSEA = 0.060, SRMR = 0.073), as suggested by Brown (2006). Model-III showed significantly high loadings for all the constructs, with reduction in measurement errors in most of the items compared to Model-II. The composite reliabilities (CRs) are all satisfactory ranging from a moderate value of 0.65 for pleasure to a high value of 0.78 of self-realization. The correlations among the factors are significantly high in this final fitted model. Thus, we have retained Model-III as the most plausible model of CASP for the small sample under study. Henceforth called the CASP-16 Brazil.

Socio-demographic characteristics may exert a direct influence on overall QoL. The data in table 5 show mean values for those in different socio-economic groups. Women reported higher average QoL scores. However the difference was not statistically significant. Interestingly the data suggest that older people who are unmarried and those who live alone are more likely to report higher levels of QoL. However this difference is only statistically significant for living along compared to living with others. The results for socio-economic position are in the expected direction. Those with a higher level of education and higher family income are more likely to report good QoL. However the differences were only statistically significant for income.

**Table 5:** Socio-demographic characteristics of those with above and below average CASP-16 scores.

	Mean	Standard deviation	p
Married	32,39	9,54	0,290
Not married	34,65	9,87	
At least elementary education	33,00	10,17	0,442
Above elementary edcuation	34,63	9,22	
Lives alone	40,23	5,83	0,008
Lives with others	32,57	9,87	
Female	35,35	10,1	0,098
Male	31,88	9,1	
Income level ≤1	28,40	10,35	0,005
Income level 2 to 5	34,16	9,62	
Income level >5	38,39	6,53	

## Discussion

This study presents the results of the cross-cultural translation, adaptation, and validation of an increasingly well used QoL scale into Brazilian-Portuguese. The CASP-16 Brazil demonstrated good psychometric properties and good internal consistency. Moreover, the CASP-16 Brazil questions were easily understood by the older respondents. The results are in line with those found by the Hyde and colleagues [17] when developing the original English version of CASP-19. Overall this suggests that, despite being developed for use within a different cultural context, the measure is able to detect the increasingly common, universal, etic dimensions to growing older in today's globalized environment.

However there are some differences from the original psychometric properties of the scale. The Pleasure domain presented the lowest alpha coefficient, meaning that it might be the domain which least contributed to the scale. However, despite variability in the responses, it has still proved to be an important component to the scale [47]. There are a number of possible reasons for these results. In cultural terms Pleasure may have been interpreted by Brazilians in this study as one of the meanings of satisfaction, the items “*I enjoy being in the company of others”* and *“On balance, I look back on my life with a sense of happiness*”, as it usually is in this country. This may have generated a weaker association. However the association is still quite acceptable (r = 0.63) compared to those found by other studies which have used the CASP-19 [22], [48]. There might also be some methodological issues which have impacted on these findings. The CASP-16 Brazil was administered in face-to-face interviews, whereas in the original Boyd-Orr follow up study, in ELSA and in SHARE it is collected by self completion [17], [22], [23]. However other studies such as Bowling and Stenner's [49], have used it in face-to-face interviewers and not reported any mode effects.

The socio-demographic characteristics of our sample are similar to those from other studies that have used the CASP-19 as far as gender is concerned [30], [50]. On the other hand, the majority of older people in Netuvelís study lived alone whereas those in Brazil usually live in family houses where there are various generations from the same family. In our case, the net income of the whole family increases providing better care and economic safety for older people, whereas in England many older people receive sufficient income to independently face the costs of living. It is possible that these cultural and economic differences may affect the quality of life in a cross-cultural study, but it is unlikely that it may affect the validation of the scale in Brazil. Hopefully future studies could include the CASP-16 Brazil in cross-national analysis of measurement invariance to examine whether factor structures differ between countries and therefore advance our knowledge about whether these are universal or culturally specific domains of QoL in later life. Yet to the best of our knowledge there are no published studies that have assessed cross-national measurement invariance in the CASP.

There are some limitations to this study that need to be acknowledged. This is a translation and validation study of the CASP-19 and the results on the scores should not be viewed as representative of the general Brazilian population. The study only covered a representative site of the city of Recife and cannot draw any comparisons with what might be found in the countryside. Also some data, such as information on income, were self-reported rather than by asking for documents. This might lead to an under-reporting of low incomes within the sample. The sample size, only 87 participants, was also rather small. However it is comparable to other QoL validation studies [11], [51]–[53].

CASP-16 Brazil has proved to be of easy application, comprehension and interpretation in evaluating QoL amongst older Brazilians. This validation study of the CASP-16 Brazil provides Portuguese-speaking countries with a good scale to measure QoL in later life. Hopefully future studies in these countries, with larger samples, will employ the measure when assessing the impact of Public Health services and socio-demographic profiles on QoL as well as those studies that focus on normal aging and cross-cultural comparisons.
